# iPAINT: a general approach tailored to image the topology of interfaces with nanometer resolution†
†Electronic supplementary information (ESI) available: Fig. S1–S8. See DOI: 10.1039/c6nr00445h



**DOI:** 10.1039/c6nr00445h

**Published:** 2016-03-30

**Authors:** A. Aloi, N. Vilanova, L. Albertazzi, I. K. Voets

**Affiliations:** a Institute for Complex Molecular Systems , Eindhoven University of Technology , Post Office Box 513 , 5600 MD Eindhoven , The Netherlands . Email: lalbertazzi@ibecbarcelona.eu; b Laboratory of Macromolecular and Organic Chemistry , Department of Chemical Engineering and Chemistry , Eindhoven University of Technology , Post Office Box 513 , 5600 MD Eindhoven , The Netherlands; c Laboratory of Physical Chemistry , Department of Chemical Engineering and Chemistry , Eindhoven University of Technology , Post Office Box 513 , 5600 MD Eindhoven , The Netherlands . Email: i.voets@tue.nl

## Abstract

Understanding interfacial phenomena in soft materials such as wetting, colloidal stability, coalescence, and friction warrants non-invasive imaging with nanometer resolution. Super-resolution microscopy has emerged as an attractive method to visualize nanostructures labeled covalently with fluorescent tags, but this is not amenable to all interfaces. Inspired by PAINT we developed a simple and general strategy to overcome this limitation, which we coin ‘iPAINT: interface Point Accumulation for Imaging in Nanoscale Topography’. It enables three-dimensional, sub-diffraction imaging of interfaces irrespective of their nature *via* reversible adsorption of polymer chains end-functionalized with photo-activatable moieties. We visualized model dispersions, emulsions, and foams with ∼20 nm and ∼3° accuracy demonstrating the general applicability of iPAINT to study solid/liquid, liquid/liquid and liquid/air interfaces. iPAINT thus broadens the scope of super-resolution microscopy paving the way for non-invasive, high-resolution imaging of complex soft materials.

## Abstract 2

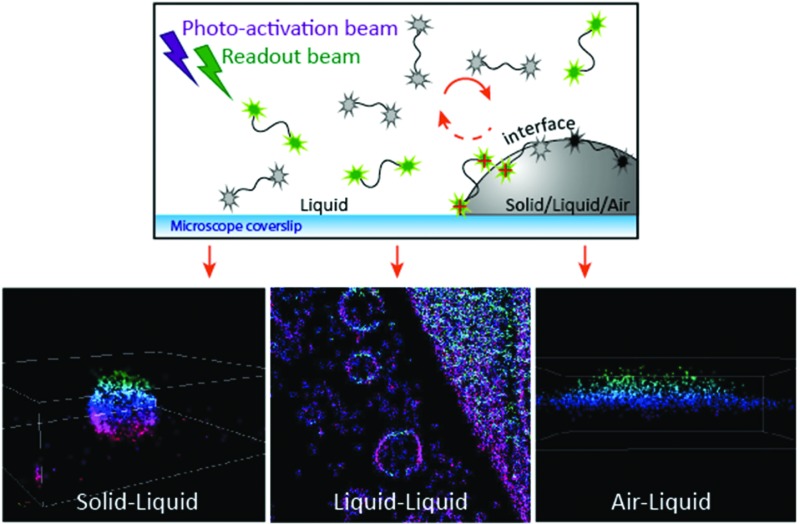
iPAINT enables three-dimensional super-resolution imaging of soft and deformable interfaces in nanomaterials without the need of covalent labelling.

## Introduction

Interfaces play an essential role in physical, biological and chemical processes, ranging from colloidal stability, energy conversion, and phase-transfer catalysis to signal-transduction, molecular recognition, and molecular transport across membranes. This is a direct consequence of their ubiquitous presence, especially in nanostructured materials with high surface-to-volume ratios due to the small dimensions of the building blocks. In the last decade super-resolution microscopy emerged as an attractive technique complementary to X-ray diffraction, electron microscopy (EM), and atomic force microscopy (AFM) to study interfaces with nanometer resolution in 3D.^1–4^ It is particularly suited for dynamic, soft materials where minimal sample perturbation is essential and differences in electron density are small. Nowadays, point accumulation for imaging in nanoscale topography (PAINT),^5^ photo-activated localization microscopy (PALM),^2^ stochastic optical reconstruction microscopy (STORM),^4^ and other single-molecule localization methods^6^ are fundamental techniques to study the morphology and dynamics of living matter.^7^ Recent STORM experiments unravelling the self-assembly mechanism and architecture of complex synthetic molecular systems^8,9^ demonstrate that super-resolution microscopy also offers unique insights into man-made materials.

Most sub-diffraction imaging methods rely on covalent labeling with fluorescent markers that can be photo-activated or blink stochastically. Dyes with suitable photophysical properties equipped with a functional group for direct coupling to the object under consideration are selected and subsequently the label density is tuned to optimize object reconstruction.^2^ PAINT on the other hand relies on non-covalent labeling, exploiting a continuous and reversible targeting of the object by freely diffusing fluorophores.^5^ In a pioneering study, Sharanov *et al.* imaged lipid vesicles by PAINT using Nile red, which fluoresces only in hydrophobic environments.^5^ Thus, probes immobilized in the lipid bilayer start to fluoresce, a diffraction-limited single-molecule image is acquired, and eventually fluorescence drops to zero as the dyes photobleach and/or dissociate from the vesicle. Subsequently, Giannone and coworkers developed uPAINT to study the structure and dynamics of membranes *via* labeling of specific membrane-bound biomolecules with a fluorescently tagged ligand.^10^ Shortly after, DNA-PAINT was developed to realize three-dimensional, multi-color, sub-10 nm imaging of DNA nanostructures and proteins with better control over the binding specificity and dissociation kinetics of the probes.^11,12^

PAINT-based techniques have been rapidly adopted as an essential research tool throughout biology and biophysics, but still remain scarcely applied in soft matter and materials science. A major hurdle for the widespread application in colloid and interface science are the stringent requirements of having hydrophobic domains (PAINT) or incorporation of specific ligand/receptor pairs (uPAINT, DNA-PAINT). To overcome these limitations, we developed PAINT further into what we coin ‘iPAINT’, which is short for interface Point Accumulation for Imaging in Nanoscale Topography. This new approach enables visualization of solid/liquid, liquid/liquid, and liquid/air interfaces with nanometer resolution in 3D irrespective of their surface chemistry *via* continuous non-covalent labeling during imaging. The latter is essential for complex interfaces that cannot be labelled directly through site-specific covalent coupling of a dye, such as emulsions, foams, and crystals like ice.

**Fig. 1:**
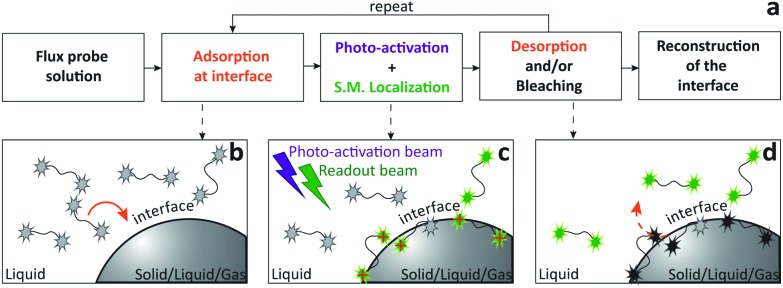
Flow chart of iPAINT super-resolution microscopy. (a) A typical iPAINT experiment commences with probe injection followed by probe adsorption, photo-activation, excitation, single-molecule (S.M.) localization, bleaching, and desorption until the object of interest is reconstructed in a final step of image analysis. (b) Upon injection of a PEG552 solution into the chamber, probes (all in the dark state) accumulate at the interface. (c) UV laser light photo-activates a limited amount of probes all of which are subsequently excited by visible laser light. Individual excited dyes immobilized at the interface are localized (red crosses). (d) Immobilized dyes bleach and/or exchange with probes in the reservoir. This repetitive sequence of events (b–d) results in continuous non-covalent labeling of the interface.

A flow chart of a typical iPAINT experiment is given in Fig. 1a. The crucial element is the presence of a large reservoir of polymers end-functionalized with photo-activatable probes (PEG552). While most of these macromolecules freely diffuse in solution, some adsorb at and desorb from the interface, allowing prolonged non-covalent labeling of the interface during imaging. PEG552 consists of a photo-activatable rhodamine analogue^13^ coupled to a poly(ethylene glycol) chain, which is well-known for reversible, non-specific adsorption onto a wide range of interfaces.^14^ At the onset of the iPAINT experiment, no fluorescence signal is collected as the probes are in the dark state (Fig. 1b). Next, low-power UV laser light (*λ* = 405 nm) photo-activates a small number of probes, while a full-power readout beam (*λ* = 561 nm) excites the activated probes (Fig. 1c). The number of fluorescent dyes in the bright state is controlled by the power of the UV laser, aiming for a probe density of several tens of excited dyes per frame of ∼1900 μm^2^. Single-molecule localization of dyes occurs solely at the interface (red crosses in Fig. 1c), since freely diffusing probes move too fast relative to the EMCCD camera acquisition rate. Continuous iteration of these steps allows reconstructing the interface with nanometer accuracy.

## Results and discussion

**Fig. 2:**
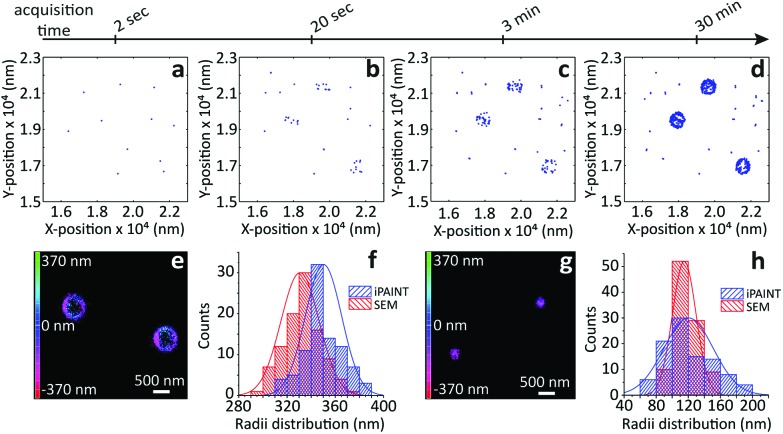
iPAINT imaging of colloidal dispersions. (a–d) iPAINT images at different time lapses after 10^2^, 10^3^, 10^4^, 5 × 10^4^ acquired frames. Blue dots correspond to individual fluorescent probes localized at the silica surface, building up the reconstructed images in time of silica beads of (e) ∼330 nm and (g) ∼110 nm in radius. Color bars indicate the *z*-position ranging from 400 nm below (pink) to 400 nm above (green) focus. (f) and (h) Distribution of particle radii obtained by iPAINT (see ESI Fig. 7†) and SEM for particles of ∼330 and ∼110 in radius, respectively.

To evaluate the possibilities and limitations of iPAINT as a complementary tool to visualize interfaces with high precision, we start off by imaging aqueous dispersions of monodisperse, spherical hydrophilic silica nanoparticles, which are broadly applied as biomaterials and in food formulations, photonics, coatings, and responsive materials.^15,16^ After a time lapse of ∼30 minutes of acquisition (Fig. 2a–d), the final reconstructed 3D iPAINT images of beads of ∼330 and ∼110 nm in radius are obtained as summation of localizations of more than 10^6^ single molecules collected in 50 000 frames. The projection on the *x*–*y* plane of each single-molecule localization on the bead surface is depicted in Fig. 2e and g. The size and shape of individual nanoparticles are clearly resolved, even though their dimensions are below the diffraction limit (∼250 nm). For benchmarking purposes, we compare the particle size distributions of >100 colloids obtained by 3D iPAINT and SEM in Fig. 2f and h (ESI Fig. 2 and 7†). We find excellent agreement for both particle sizes with mean radii differing only by less than 5%: <*R*>_iPAINT_ = 350 ± 15 nm *vs*. <*R*>_SEM_ = 332 ± 18 nm and <*R*>_iPAINT_ = 118 ± 26 nm *vs*. <*R*>_SEM_ = 115 ± 14 nm. Next, we imaged stearyl-alcohol coated (hydrophobic) colloids to assess whether iPAINT is amenable to dispersions irrespective of their wettability. Satisfactorily, we again obtain well-reconstructed 3D iPAINT images with comparable resolution (ESI Fig. 4 and 5†).

The non-covalent labeling approach of iPAINT allows for imaging of both hydrophilic and hydrophobic interfaces with long acquisition times, about five times higher than what is typically achieved with PALM.^2^ This is because the number of single-molecule localizations is less limited by depletion of fluorescent probes due to photobleaching, as bleached dyes at the interface are continuously exchanging with new photo-activatable dyes from the large reservoir of PEG552 in solution (ESI Fig. 3†). The long acquisition time results in a high number of localizations (>10^6^) which enables selection of localizations from dyes that emit a high number of photons (>10^4^) during analysis. This improves both the reconstruction of the silica interface and the accuracy in the localization of each dye (ESI Fig. 6†).^5^

**Fig. 3:**
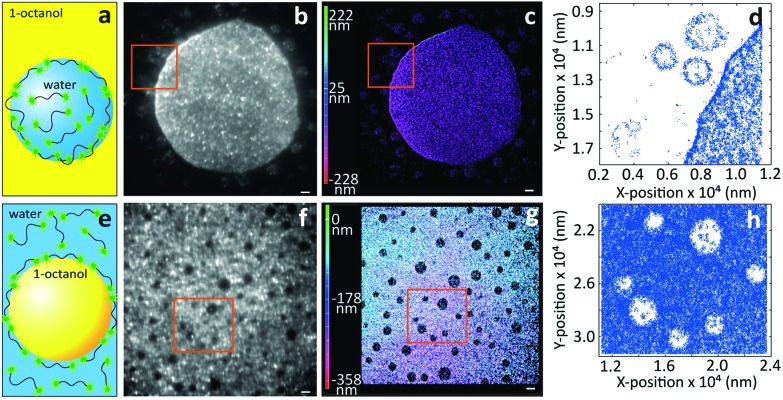
Imaging of emulsions by iPAINT microscopy. (a) Cartoon of a water droplet with PEG552 dispersed in 1-octanol. (b) Widefield and (c) iPAINT images of a W/O emulsion (scale bar 2 μm). (d) Zoom-in of iPAINT image in (c) depicts three aqueous nanodroplets which are less than 300 nm apart. (e) Cartoon of an 1-octanol droplet dispersed in a PEG552 solution. (f) Widefield and (g) iPAINT images of an O/W emulsion (scale bar 2 μm). (h) Zoom-in of iPAINT image in (g) depicts oil nanodroplets with *R* < 600 nm.

Next, we turn to three-dimensional, non-invasive, high-resolution imaging of emulsions, which is a challenging task since droplets are dynamic and their interface is deformable under applied pressure. To this end we prepared model water-in-oil (W/O) and oil-in-water (O/W) emulsions from 1-octanol and water, which are used as a model to study the partitioning of species from water into soil^17^ and to mimic the adsorption of molecules into living tissues.^18^ To circumvent Gaussian blurring due to diffusion, droplets need to be immobilized onto glass coverslips. Fig. 3 shows a comparison between widefield and iPAINT imaging of W/O (Fig. 3a–d) and O/W (Fig. 3e–h) emulsions. The oil phase in Fig. 3b and f appears dark, whereas the aqueous phase is bright, since PEG552 adsorbs onto the oil/water and coverslip/water interfaces. Only large droplets are clearly distinguishable by widefield microscopy, while iPAINT resolves nanometer- to micrometer-sized oil and water droplets. The reservoir with PEG552 probes in the W/O emulsion (∼10^4^ molecules in a 1 μm diameter droplet) is too small for a neat reconstruction of the interface of sub-diffraction-sized nanodroplets, but it is sufficient to identify them (Fig. 3d). By contrast, the larger aqueous reservoir in O/W emulsions contains sufficient PEG552 for full reconstruction of the oil/water interface of small droplets.

**Fig. 4:**
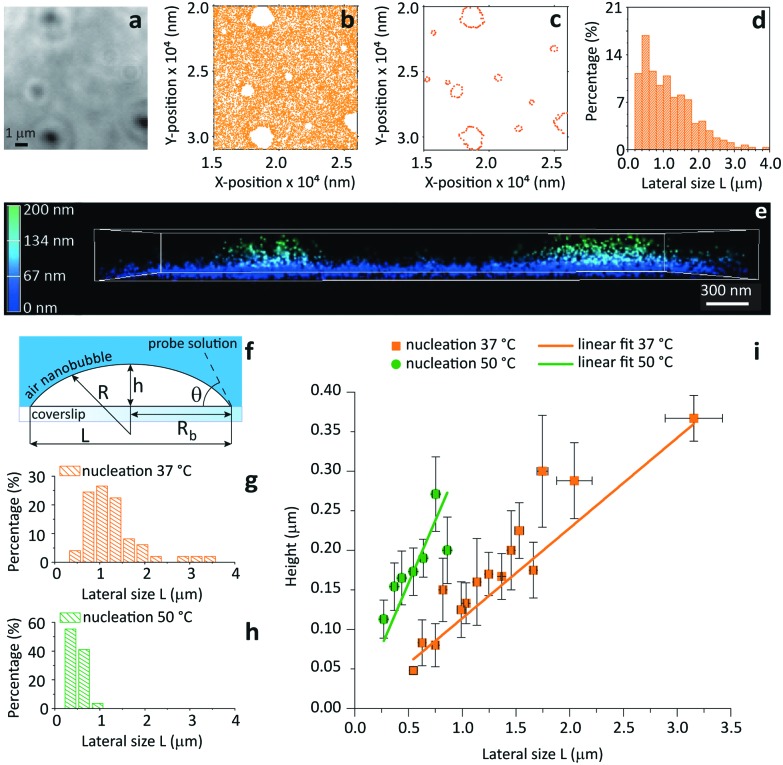
Contact angle measurements of individual nanobubbles by iPAINT. (a) Brightfield imaging of air bubbles nucleated on a glass coverslip by solvent-exchange.^19^ (b) iPAINT imaging of the same region as in (a) reveals air nanobubbles smaller than the diffraction limit. (c) Identification of the contours of single air nanobubbles (for further details see ESI†). (d) Lateral size distribution of nanobubbles shown in (c). (e) 3D iPAINT imaging of two nanobubbles. Single-molecule localizations at the air/water interface are color-coded according to their distance relative to the coverslip from blue (0 nm) to green (200 nm); adsorption at the water/coverslip interface is visible as a non-negligible background of localizations throughout the coverslip at ∼0 nm. (f) Cartoon of an immobilized air bubble indicating the contact angle, *θ*, the bubble height, *h*, the base radius, *R*_b_, the radius of curvature, *R*, and the lateral size, *L*. (g–h) Lateral size distributions of air nanobubbles nucleated at 37 °C and 50 °C, respectively. (i) Bubble height as a function of lateral size for 37 °C and 50 °C. The error bars represent the standard deviation. Average contact angles of *θ*_37 °C_ = 13° ± 0.7° and *θ*_50 °C_ = 35° ± 2.6° were determined fitting the data to eqn (1) (*R* ^2^ = 0.98 for both nucleation temperatures).

As a final test for the general applicability of iPAINT for interface imaging we visualize air nanobubbles, so far accomplished only by AFM.^19^ Nanobubbles are an active area of physico-chemical research as they impact a range of interfacial phenomena including molecular adsorption, thin film rupture, and surface corrosion.^20^ Fig. 4 shows brightfield (BF) and iPAINT images of air nanobubbles, nucleated at 37 °C on a glass coverslip *via* alcohol-water exchange.^19^ Microbubbles are visible in the BF image in Fig. 4a, but their size cannot be determined accurately. Conversely, the distribution of lateral bubble sizes *L* is readily determined from the bubble contours identified by iPAINT (Fig. 4b–d, ESI Fig. 8†), unveiling sub-diffraction-sized air bubbles. Analogous to the O/W emulsions, PEG552 adsorbs at the relevant interface as well as on the glass coverslip in contact with water, which means that the air bubbles are the areas without single-molecule localizations in the iPAINT image (Fig. 4b).

The height *h* and lateral size *L* = 2R_b_ of nanobubbles nucleated at 37 °C and 50 °C are determined from 3D iPAINT images (Fig. 4e–h). These two nucleation temperatures were selected to compare our measurements directly with AFM data from others and investigate whether temperature alters bubble morphology.^21^ In accordance with previous work by others, we obtain smaller mean lateral sizes and lateral size distributions for bubbles nucleated at 50 °C (Fig. 4g and h), presumably due to an increased mobility of gas molecules at elevated temperatures.^21^ We find the non-equilibrium contact angles *θ*_37°C_ = 13° ± 0.7° and *θ*_50°C_ = 35° ± 2.6° (Fig. 4i) using the following simple equation:^22^
1
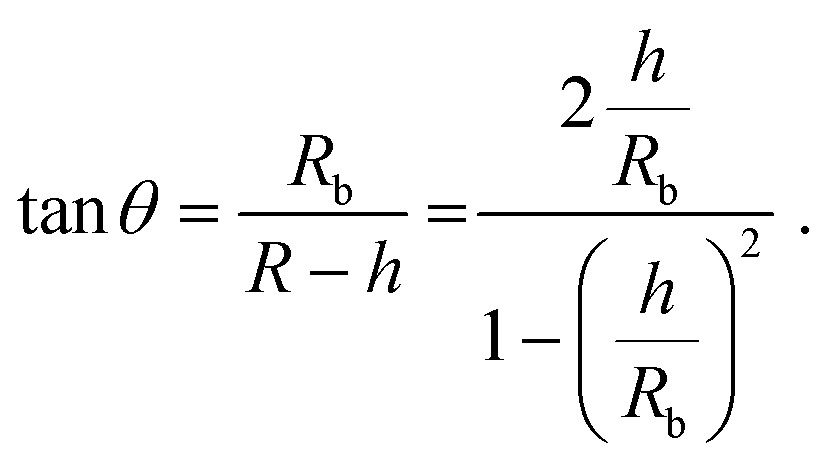



Our findings clearly confirm the influence of the substrate temperature during nucleation on bubble morphology.^23–25^ Gratifyingly, iPAINT thus offers a complementary non-invasive method to investigate the morphology and contact angle of individual air nanobubbles. This enables an independent verification of AFM results, which has been long sought-after since perturbation of the nanobubbles by the AFM tip could lead to an underestimation of the actual contact angles.^26^

## Experimental

### Materials and methods

#### Synthesis

Poly(ethylene glycol) bis(amine) MW 20 kDa (PEG) was purchased from Sigma Aldrich; an *N*-hydroxysuccinimide ester activated rhodamine analogue (Cage552) designed for photo-activation localization microscopy was purchased from Abberior®. 1 mg of PEG was dispersed in 1 mL of 0.1 M sodium bicarbonate buffer at pH 8.5 to which 20 μL of a 10 mM solution of Cage552 in DMSO was added. The reaction mixture was stirred overnight in the dark at room temperature and subsequently purified by dialysis (Spectra/Por®7 dialysis membrane, pre-treated RC tubing, molecular weight cutoff: 8 kDa) to remove unreacted dye molecules, which was confirmed by gel permeation chromatography (ESI Fig. 1†).

#### Microscopy

iPAINT images were acquired using a Nikon N-STORM system equipped with ∼55.2 mW (*λ* = 561 nm) and ∼17.9 mW (*λ* = 405 nm) laser lines configured for total internal reflection fluorescence (TIRF) imaging. The excitation inclination was tuned to maximize the signal-to-noise ratio. Fluorescence was collected by means of a Nikon 100×, 1.4NA oil immersion objective and passed through a quad-band pass dichroic filter (97335 Nikon). All time-lapses were recorded onto a 128 × 128 pixel region (pixel size 170 nm) of an EMCCD camera (ixon3, Andor) at a rate of 47 frames per s. Unless stated otherwise, 2 × 10^4^ frames were acquired in each experiment, while the Cage552 moieties were photo-activated with a 405 nm UV laser (0.5% power) and excited with a 561 nm laser (100% power). Single molecule localization movies were analyzed with NIS-element Nikon software. 3D iPAINT measurements were performed using the astigmatism method.^27^ The *z*-position in 3D iPAINT experiments on dispersions and bubbles is computed using a calibration curve made with fluorescent TetraSpeckTM microspheres (*R* = 50 nm, Life-technologies, Molecular Probes®) that relates the ellipticity of the fluorescence signal of single molecules to their *z*-position.

#### Sample preparation

Sample chambers consist of a coverslide (Menzel Gläser, 76 × 26 mm, thickness 1 mm) onto which a coverslip (Menzel Gläser, no. 1.5, 24 × 24 mm, thickness 170 μm) is glued with double-sided tape. Prior to assembly of the chamber, the coverslip is cleaned to remove impurities and reduce background fluorescence as follows: it is consecutively immersed and 10 minutes sonicated in acetone, isopropanol and MilliQ water (18.2 MΩ) after which it is dried with a N_2_ stream.

#### Colloidal dispersions

Hydrophilic (plain) silica colloids with low polydispersity were synthesized using the Stöber method (see ESI†).^28^ Hydrophobic silica beads were obtained by surface-functionalization of these plain beads with *n*-octadecyl alcohol. Size and polydispersity of silica beads were determined by SEM (ESI Fig. 2†). iPAINT samples were prepared by application of a few drops of the colloidal dispersion on a coverslip, followed by drying in N_2_ stream prior to closure of the sample chamber, after which a freshly prepared 50 μM PEG552 solution was fluxed into it.

#### Emulsions

Water-in-oil and oil-in-water emulsions (10 wt% dispersed phase) were prepared by direct mixing of a 50 μM PEG552 solution with chromatographic grade 1-octanol from Sigma-Aldrich, followed by 5 minutes sonication. Hydrophobic coverslips were made for iPAINT experiments on O/W emulsions to immobilize the oil droplets. To this end, hydrophilic coverslips were first cleaned by piranha etching and extensively rinsed with MilliQ-water, and subsequently silanized as follows. Coverslips were first incubated for 15 minutes in 5% dimethyl-dichlorosilane in heptane (Sigma Aldrich), then cleaned with heptane, blow-dried under N_2_ flow and finally dried at 60 °C for two hours.

#### Nanobubbles

Air bubbles were nucleated at 37 °C and 50 °C using the solvent exchange protocol.^22^ First, the sample chamber was assembled and heated to 37 °C or 50 °C. Then, 2-propanol is injected and subsequently replaced by an aqueous PEG552 solution, which induces nanobubble nucleation. Imaging is carried out at room temperature to avoid water evaporation.^22^

## Conclusions

In summary, we have introduced a powerful new super-resolution approach called iPAINT, tailored to investigate interfaces of different nature through continuous non-covalent labeling during imaging. iPAINT is a generic method able to super-resolve interfaces in three-dimensions in complex soft materials, such as dispersions, emulsions, and foams. The key innovation is simple: a continuous exchange at the interface between surface-bound and freely diffusing polymer chains end-functionalized with a photo-activatable moiety. This novel approach broadens the scope of PAINT to colloid and interface science, food science, soft matter physics, and nanotechnology. We anticipate that iPAINT will find widespread use in these areas, particularly for non-invasive 3D imaging of the topology of soft and dynamic interfaces, such as droplets, bubbles, and ice crystals.
