# Supplementary material for: iPAINT: a general approach tailored to image the topology of interfaces with nanometer resolution
Source: Nanoscale. 2016 Mar 30;8(16):8712–6. doi: 10.1039/c6nr00445h (PMC5050559; doi:10.1039/c6nr00445h)
Supplement: Supplementary file 1 [file NR-008-C6NR00445H-s001.pdf]

Electronic Supplementary Material (ESI) for Nanoscale.
This journal is © The Royal Society of Chemistry 2016

iPAINT: a general approach tailored to image the topology of interfaces with nanometer resolution

– supplementary information –

Antonio Aloi[a,b], Neus Vilanova[a,b], Lorenzo Albertazzi*[c], Ilja K. Voets*[a,b,d]

a Institute for Complex Molecular Systems, Eindhoven University of Technology, Post Office Box 513, 5600 MD Eindhoven, Netherlands.

b Laboratory of Macromolecular and Organic Chemistry, Department of Chemical Engineering and Chemistry, Eindhoven University of Technology, Post Office Box 513, 5600 MD Eindhoven, Netherlands.

c Current address: Nanoscopy for nanomedicine group, Institute for Bioengineering of Catalonia (IBEC), C. Baldiri Reixac 15-21, 08028 Barcelona, Spain. E-mail: lalbertazzi@ibecbarcelona.eu

d Laboratory of Physical Chemistry, Department of Chemical Engineering and Chemistry, Eindhoven University of Technology, Post Office Box 513, 5600 MD Eindhoven, Netherlands. E-mail: i.voets@tue.nl

This document includes:

- **Figure S1 to S8**
- **Additional GPC and SEM measurements**
- **Image analysis procedures**
- **References**

Characterization of polymer chains labelled with a photo-activatable moiety

Fig. S1 shows the Gel Permeation Chromatography (GPC) spectra of the photo-activatable probe (PEG552) and of the free dye (Cage552). The latter elutes at 11.5 min. The absence of a peak at 11.5 min in the PEG552 spectrum demonstrates the absence of free dye in the dye solution for iPAINT imaging.

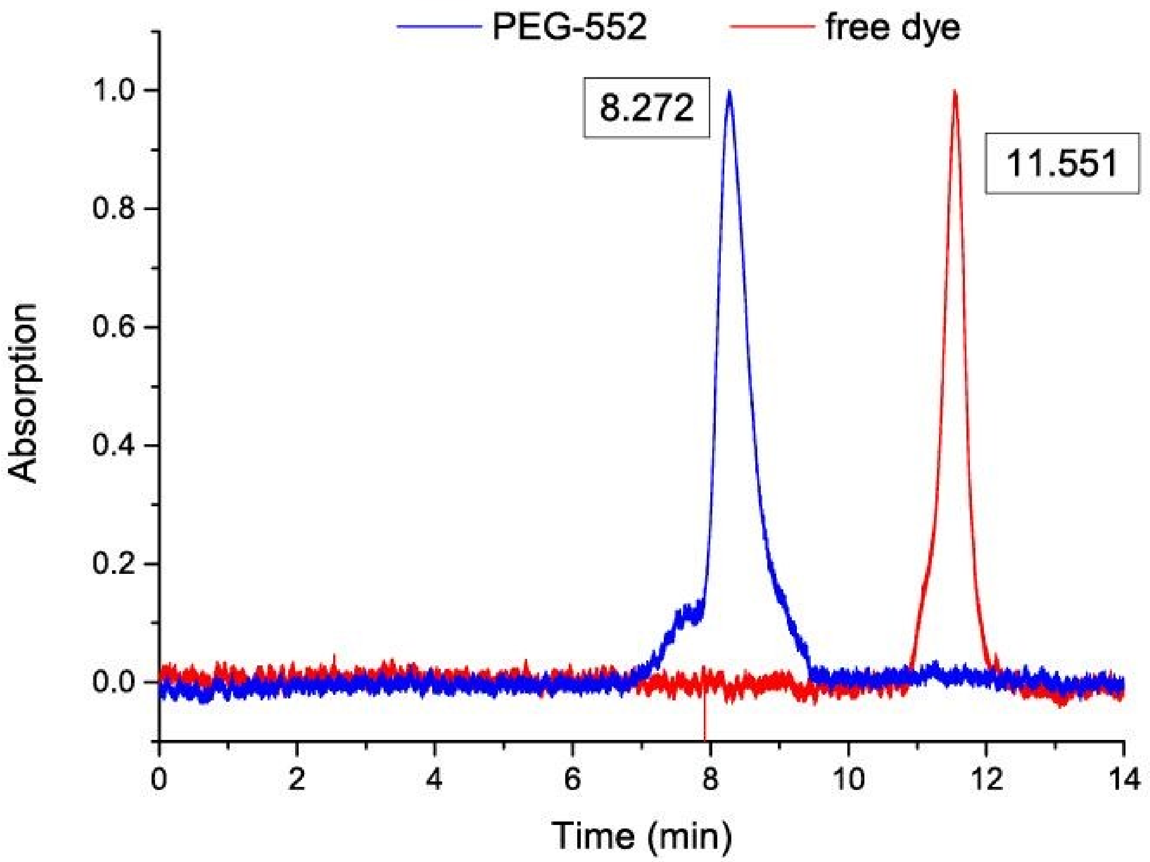

Fig. S1 Characterization of photo-activatable probes for iPAINT microscopy. GPC chromatograms of aqueous solutions of PEG552 (5×10^{-5} M) and the free dye Cage552 (10^{-6} M) depicted in blue and red, respectively.

Synthesis and SEM characterization of silica colloids

Silica colloids of low polydispersity were synthetized using a Stöber-based method.[1-3] Briefly, 6.4 µL of (3-Aminopropyl)triethoxysilane (APTES) were mixed with 33.5 mL of ethanol and 2.8 mL of ammonia (25% in water). 2.5 mL of this solution were added to a mixture of 25 mL of ammonia (25% in water) and 250 mL of ethanol. 1.6 mL of tetraethylortosilicate (TEOS) were then added all at once and the final mixture was stirred for 5 hours. Subsequently, 1.75 mL of TEOS were added and the reaction mixture was stirred overnight under an argon atmosphere. Following this procedure, particles of ~110 nm in radius were obtained. Such particles were further used as seeds to synthesize bigger particles by means of growing a silica shell onto them. To do so, in a round-bottom flask equipped with 2 inlets, 51 mL of ethanol, 17 mL of water, 3.4 mL ammonia (25% in water) and 4 mL of the

seed suspension (solid content 0.0136g seeds/mL of suspension) were mixed. Through one of the inlets, a mixture of 5 mL of TEOS with 10 mL of ethanol was pumped with a peristaltic pump at 1.7 mL/hour. Through the other inlet a second mixture consisting of 1.34 mL ammonia (25% in water), 10.25 mL of ethanol and 3.4 mL of water was also pumped with the same flow rate. After all the reactants were added, the resulting suspension of silica colloids (of ~330 nm in radius approximately) was washed and stored in ethanol.

Silica particles were characterized by SEM using a FEI Quanta 600F ESEM. Fig. S2 shows the SEM images of the big and small colloids. The insets depict the size distribution obtained from image analysis of more than 100 particles, using Image J: $R_{mean} = 115 \pm 14$ nm for the small and $R_{mean} = 332 \pm 18$ nm for the big colloids.

Hydrophobic silica colloids were prepared by functionalizing plain silica colloids with stearyl alcohol.[4] 10 mg of dried plain silica colloids were dispersed by sonication in 1 mL of ethanol together with 40 mg of stearyl alcohol. Afterwards, the ethanol was totally evaporated with a steady stream of argon. The flask, equipped with a magnetic stirrer, was heated up to 180 °C for 6 hours under continuous stirring and in an argon atmosphere. The resulting particles were thoroughly washed with chloroform and dried overnight at 70 °C in vacuum. For the iPAINT experiments, hydrophobic silica colloids were finely re-suspended in ethanol using a sonication bath.

All chemicals were from Sigma-Aldrich and used without further purification.

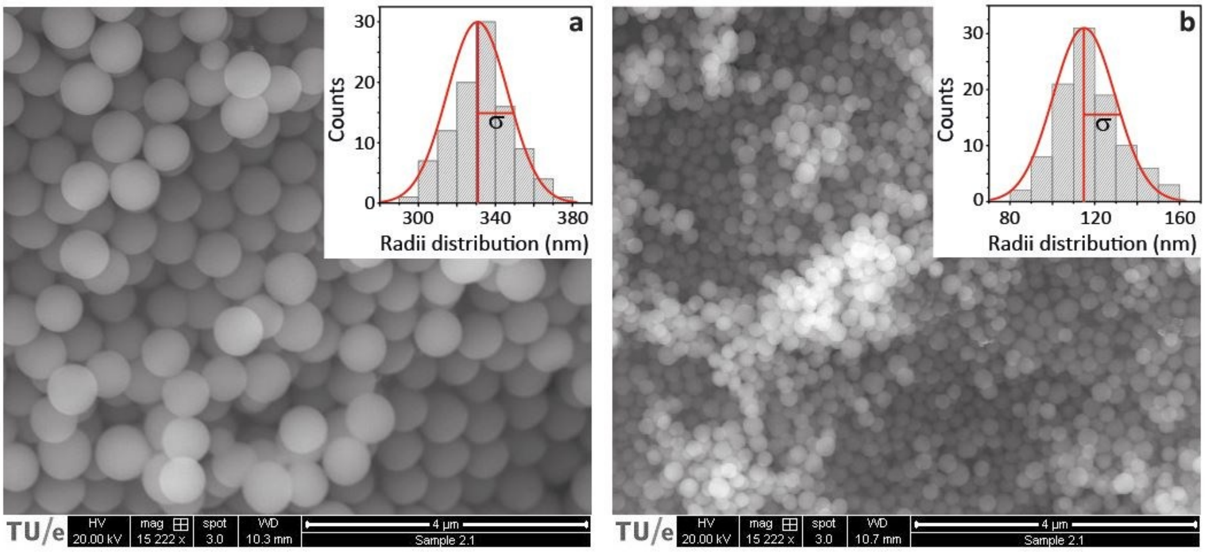

Fig. S2 Characterization of silica colloids. (**a**) and (**b**) SEM pictures of plain silica beads of ~330 nm and ~110 nm in radius, respectively. The insets illustrate the size distribution of the two batches measured for over 100 beads.

Continuous exchange of photo-activatable probes enables long acquisition times

iPAINT enables long acquisition times - roughly five times longer acquisition times than regular super-resolution microscopy[5] - due to continuous exchange of dyes between the interface and the solution. The latter serves as a large reservoir of photo-activatable, non-bleached fluorescent probes in solution. To demonstrate the impact of dye exchange, a long acquisition time-lapse of 10^5 frames was collected in the presence of freely diffusing PEG552 chains in the sample chamber (5×10^{-5} M). Fig. S3 shows the number of localizations, normalized per bead and per acquired frame as a function of acquisition time. After a short equilibration period of few minutes, the number of localizations remains constant as a steady state is reached due to the balance between adsorption, desorption and bleaching of the fluorescent probes. During this period, the density of fluorescent probes at the interface is essentially constant and photobleaching hardly reduces the number of excited probes at the interface since bleached dyes are rapidly replenished by photo-activatable dyes from the large reservoir. This enables localization of a large number of individual probes which improves the resolution of the experiment. Upon replacing the PEG552 solution by flushing MilliQ into the chamber (dashed line in Fig. S3), which prevents the exchange during imaging, the number of localizations per bead and per frame rapidly reduces until finally all probes are bleached. This demonstrates that reversible exchange of dyes between the interface and the large reservoir of dyes in solution is essential to reach long acquisition times in iPAINT.

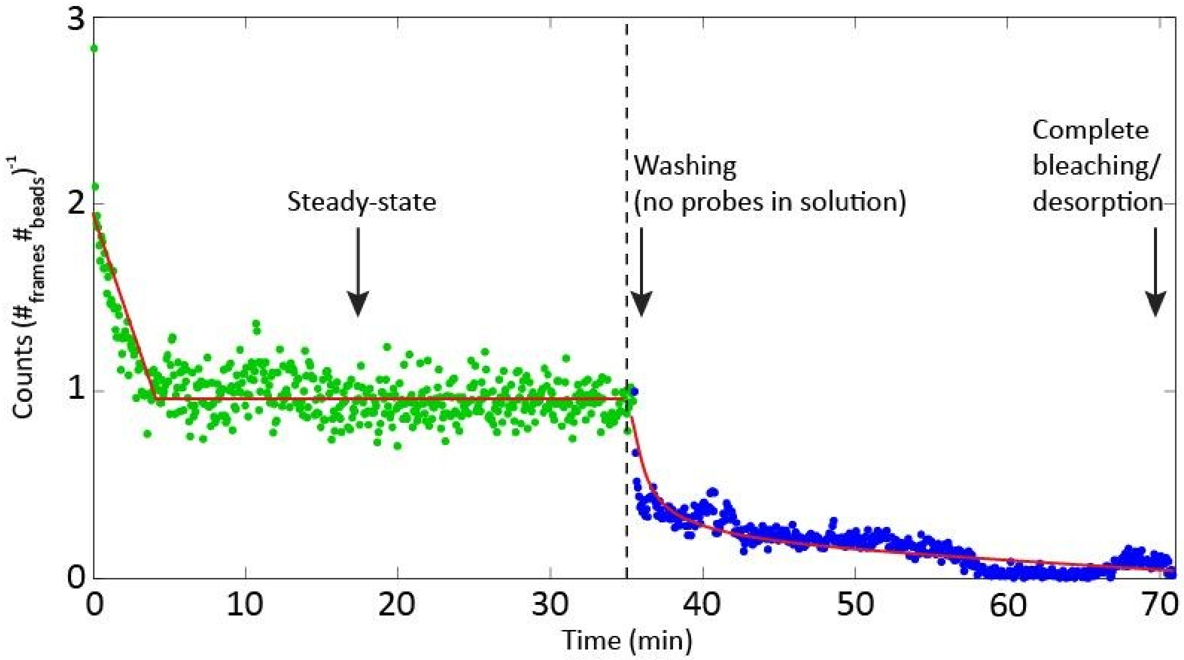

Fig. S3 Continuous exchange of photo-activatable probes enables long acquisition times in iPAINT. Number of single molecule localizations per bead and per frame *N* during a long acquisition time-lapse of 10^5 frames in total. In the presence of a large reservoir of PEG552, *N* rapidly plateaus at a constant value as a steady state is reached due to a balance between adsorption, desorption and photobleaching. After 35 min, the PEG552 solution is replaced by MilliQ, which prevents continuous exchange of dyes during imaging and thus leads to a rapid decrease in the number of localizations until finally all dyes are bleached. Lines are drawn to guide the eye.

iPAINT imaging of hydrophilic and hydrophobic colloids

Hydrophilic (plain) and hydrophobic (stearyl-coated) silica beads were imaged by iPAINT microscopy to investigate whether iPAINT can be applied to image both hydrophilic and hydrophobic solid/liquid interfaces. Both hydrophilic (Fig. S4a) and hydrophobic (Fig. S4c) silica nanoparticles were imaged in three-dimensions with ~20 nm accuracy. The particle size distributions were computed for both hydrophilic (Fig. 5Sa) and hydrophobic (Fig. 5Sb) silica colloids using 2D and 3D fitting routines on the pointcloud localizations (see image analysis section for further details). For the hydrophilic nanoparticles we obtained R_{mean_2D} = 349 ± 16 nm and R_{mean_3D} = 358 ± 18 nm and for the hydrophobic colloids we found R_{mean_2D} = 346 ± 15 nm and R_{mean_3D} = 351 ± 17 nm.

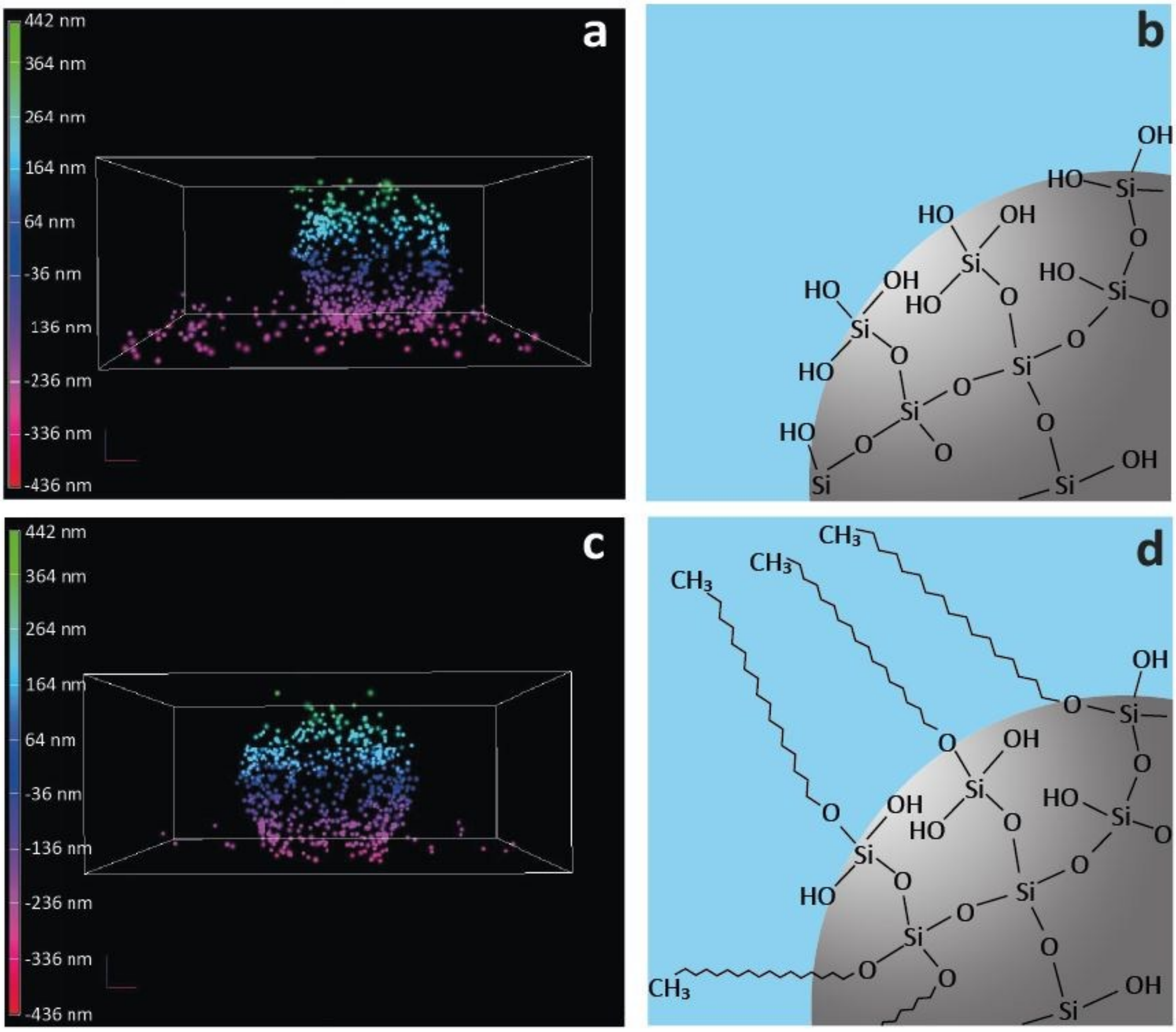

Fig. S4 3D iPAINT imaging of hydrophilic and hydrophobic nanoparticles. (**a**) and (**c**) 3D iPAINT reconstruction of a plain (hydrophilic) and stearyl alcohol-coated (hydrophobic) silica bead, respectively. (**b**) and (**d**) Cartoons representing the surface chemistry of a hydrophilic and hydrophobic silica bead, respectively.

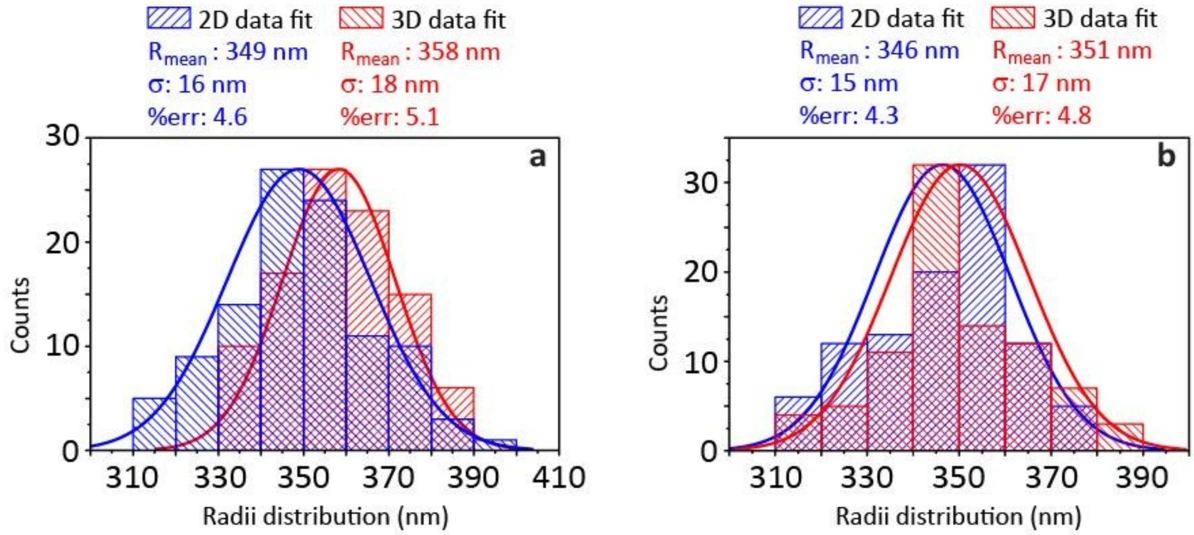

Fig. S5 Size distributions of silica colloids of hydrophilic and hydrophobic nanoparticles. Colloid size distributions from two- (blue) and three-dimensional (red) fitting routines of the localization pointcloud, reconstructing the hydrophilic (**a**) and hydrophobic (**b**) surface of silica beads.

Enhancing the accuracy of iPAINT imaging through long acquisition times

The accuracy of object reconstruction is dependent on the total number of localizations used for reconstruction as well as the accuracy of the individual single molecule localizations. The theoretical accuracy of each single molecule localization is given by:[6]

$$\sigma_{\mu_i} = \sqrt{\left(\frac{s_i^2}{N}\right) + \left(\frac{a^2/12}{N}\right) + \left(\frac{8\pi s_i^4 b^2}{a^2 N^2}\right)} \propto \frac{s_i}{\sqrt{N}} \qquad (S1)$$

where σ is the standard error to the mean in the photon distribution ($\mu = x_0; y_0$), a is the pixel size of the imaging CCD detector, b is the standard deviation of the background (including the background fluorescence emission combined with detector noise), s_i is the standard deviation of the localization distribution (in direction i, with i = x or y), and N is the number of photons gathered. The first term in equation S1 refers to the photon noise, the second term takes into account the finite size of the pixels of the detector, and the third term defines the background noise. Typically the noise term due to pixelation $\left(\frac{a^2/12}{N}\right)$ and the background noise $\left(\frac{8\pi s_i^4 b^2}{a^2 N^2}\right)$ are negligible compared to the main noise contribution which is given by the point-spread function width of the microscope, hence equation S1 can be simplified by omitting the second and the third term.

iPAINT imaging in the presence of a large reservoir of fluorescent probes enables long acquisition times, which increases the total number of localized single molecules at the interface (>10^6). This offers the possibility to not use all localizations for reconstruction, but instead to select the localizations from dyes that emit a large number of photons. Fig. S6

shows a reconstruction of hydrophilic silica colloids using all localizations (Fig. S6a) and selected localizations only (Fig. S6b). The fluorescent tags emit at least 1000 (Fig. S6a) or 11000 (Fig. S6b) photons, which gives a theoretical accuracy for each single molecule localization of 10 nm and 1-2 nm, respectively. By α-shaping[7] we obtain a 3D reconstruction of the object (Fig. S6b, S6d). Clearly, increasing the photon threshold improves the 3D reconstruction. The lower threshold of 1000 photons yields a shape that is not perfectly spherical, particularly the top of the bead is flat and enlarged. This is because the diameter of the bead approaches the maximum height that can be probed in our microscope. Gratifyingly, the higher photon threshold of 11000 photons does enable accurate reconstruction of the entire bead.

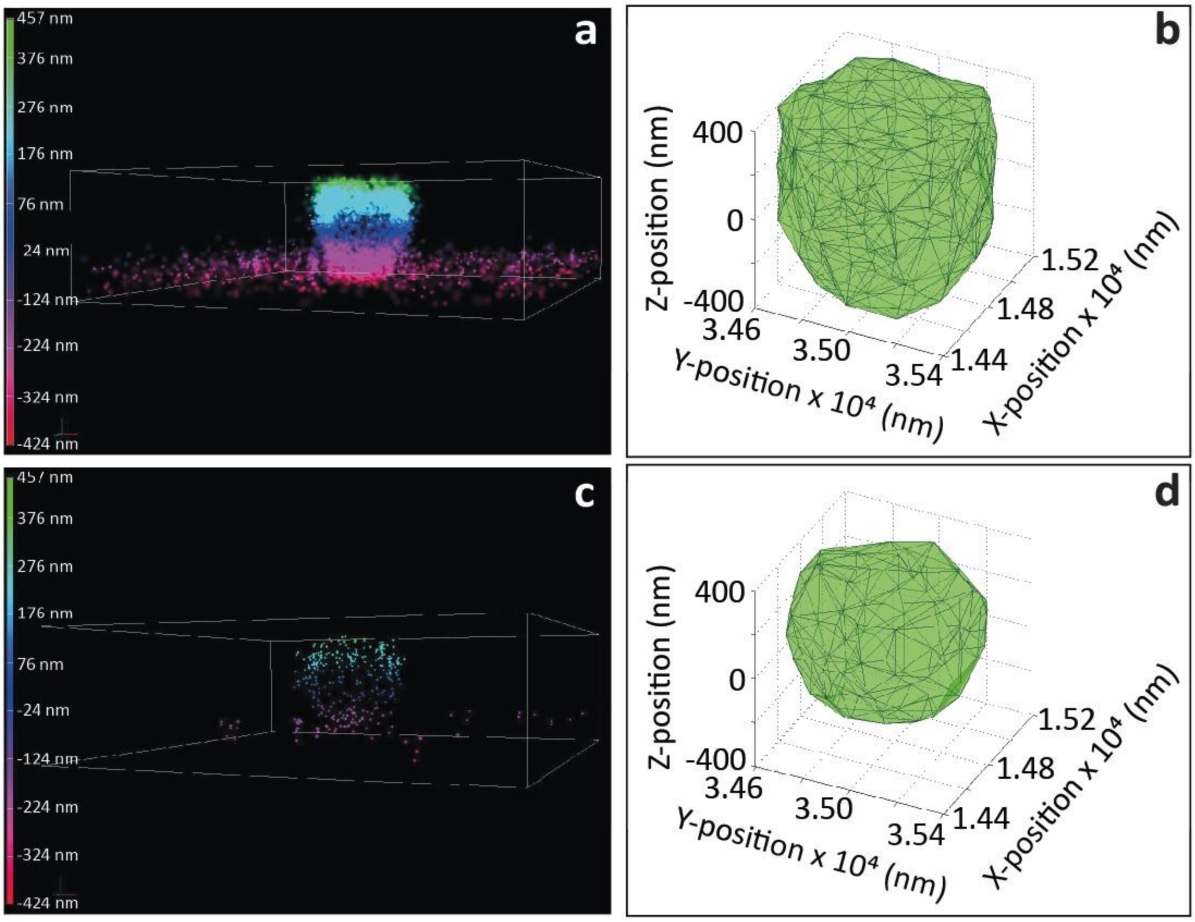

Fig. S6 Enhanced localization accuracy in iPAINT. 3D iPAINT reconstructed images of hydrophilic silica beads, ~330 nm in radius, with a photon threshold set to (**a**) 1000 and (**c**) 11000, corresponding to theoretical accuracies of 10 and 1-2 nm, respectively. (**b**) and (**d**) α-shape triangulation of the localizations shown in (**a**) and (**c**), respectively.

Image analysis

The image analysis procedure for iPAINT is outlined in Fig. S7. First, the raw image (Fig. S7a) is corrected for the background signal from fluorescent probes adsorbed on the glass coverslip. The background signal removal (implemented in the NIS element Nikon software)

is density-based: the algorithm looks for datapoints having at least *n* other datapoints within a certain range δ, and removes those localizations which do not fulfil the set parameters (*n*=10, δ=150 in Fig. S7b). Next, single beads and clustered beads are identified by eye (Fig. S7c). The single molecule localizations corresponding to each single bead are saved in a separate file for further analysis of particle size and size distribution (Fig. S7d). Mean particle sizes were determined using freeware exchange Matlab files for two- and three-dimensional fitting routines. The 2D routine utilizes the single molecule localizations corresponding to the mid-plane of the bead, taken as $\frac{z_{max}}{2}$, where z_{max} is the highest z-plane with localized single molecules. The distribution of points in this plane was fitted to a circle (Matlab exchange file “Circle Fit”) as shown in Fig. S7e, which outputs the center coordinates and the circle radius. The algorithm minimizes $\sum (x^2 + y^2 - R^2)^2$, with x and y being the coordinates of the localizations and *R* the radius of the circle. The 3D fitting routine uses the entire three-dimensional data set collected for each single bead that is identified. The localization pointcloud is fit with a Matlab exchange file “Sphere Fit” that again computes the center coordinates (x_c,y_c,z_c) and *R* (Fig. S7f). Here, $\sum \left[(x - x_c)^2 + (y - y_c)^2 + (z - z_c)^2 - R^2\right]^2$ is minimized. Fig. S5 depicts the size distributions and main radii obtained from fits of over hundreds of hydrophilic and hydrophobic beads.

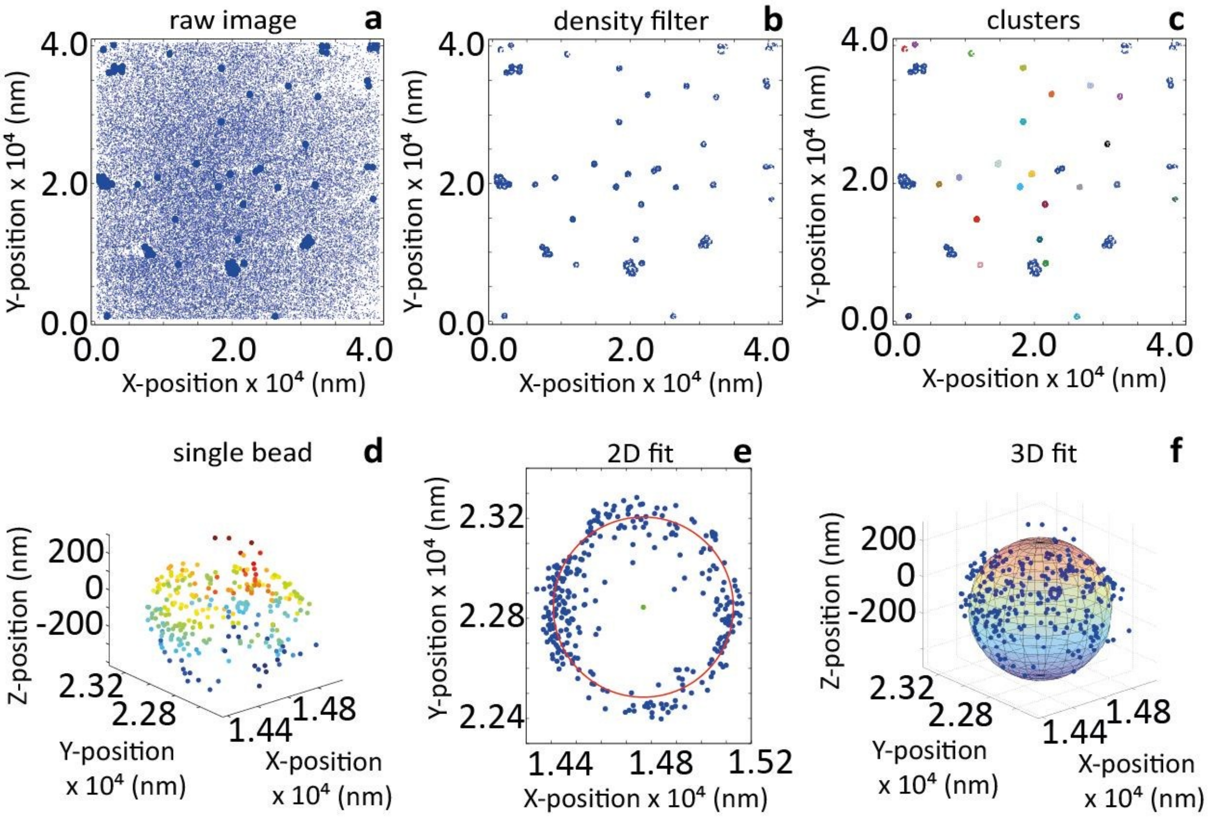

Fig. S7 iPAINT data analysis routines. Representative iPAINT image of hydrophilic silica beads of ~330 nm radius (**a**) prior, and (**b**) after background removal. (**c**) Identification of singlets; clustered beads are not taken into account in further data analysis steps. (**d**) Three dimensional iPAINT image of a single bead. (**e**) Circular fit

of localizations in the mid-plane of the bead shown in panel (**d**) to find the bead center and radius. (**f**) Three-dimensional fit of a sphere to all single molecule localizations of the entire bead shown in (**d**) to find the bead center and radius.

Non-invasive measurement of the contact angle of air nanobubbles

The contact angle of individual air nanobubbles can be determined from their height and lateral size using equation 1 in the main text.[8] To this end we utilize an α-shaping algorithm in Matlab.[7] First, the surface of the nanobubble is identified by triangulation of the coordinates of the single molecule localizations. The lateral size L is then easily computed from the boundaries of the air nanobubble on the cover slip. Next, the three-dimensional profile is divided into 80 nm thick slices, since this thickness is slightly bigger than the achieved resolution in the z-direction (Fig. S8b-e). This allows to compute the height of the nanobubbles from the difference in z-position between the lower (Fig. S8b) and the upper planes (Fig. S8e) containing single molecule localizations, such that $h = z_{max} - z_{min}$.

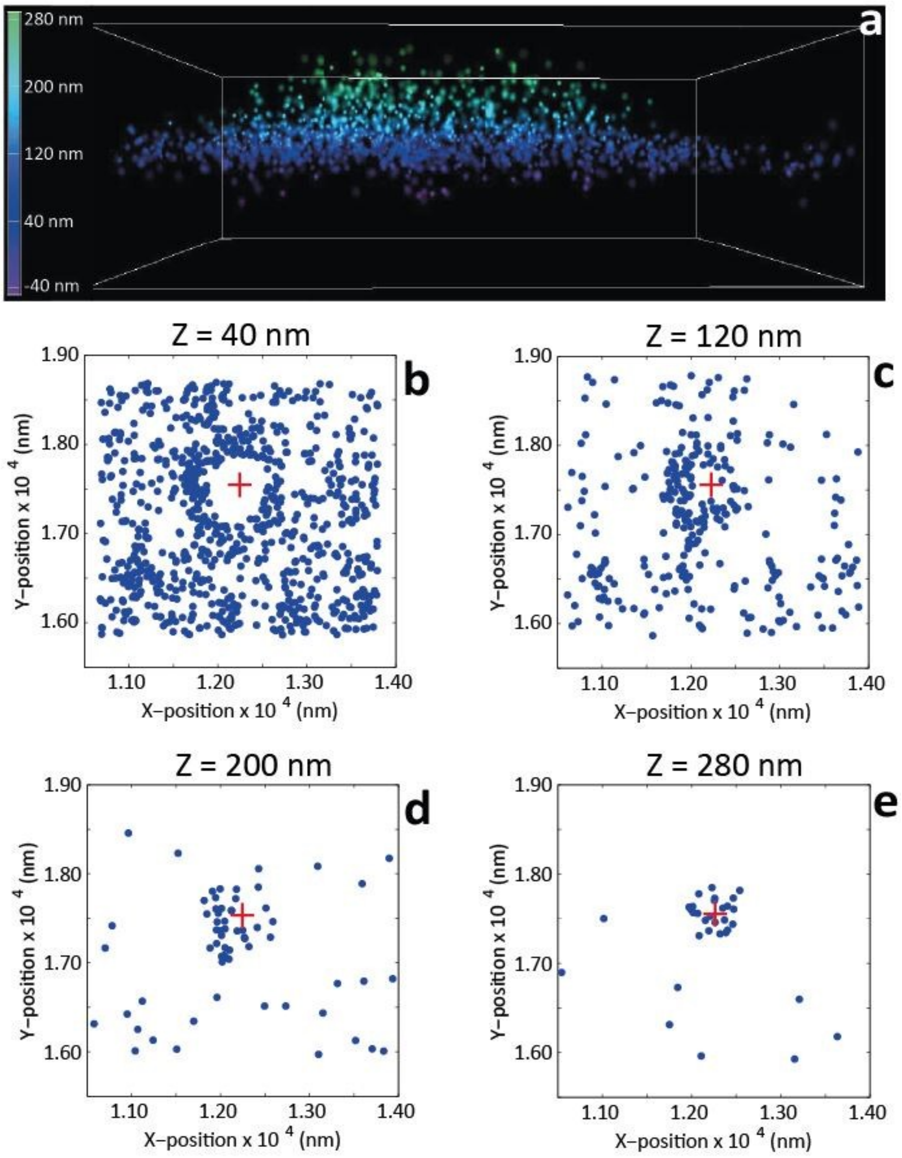

Fig. S8 Z-stack of a nanobubble. (**a**) iPAINT image of an air nanobubble in three dimensions. To compute bubble height, the image is sectioned in the z-direction into four planes of 80 nm in thickness. The lateral size of the air nanobubble is determined from the lower slice (**b**). The hollow cavity corresponding to the bubble, shrinks with increasing distance away from the coverslip (**b**-**e**). The bubble height h is taken as $h = z_{max} - z_{min}$. The red cross in (**b**-**e**) identifies the center of the nanobubble.

References

1 W. Stöber, A. Fink, E. Bohn, *J. Colloid Interf. Sci.*, 1968, **26**, 62-69.

2 H. Giesche, *J. Eur. Ceram. Soc.*, 1994, **14**, 189-204.

3 H. Wu, *Chem. Phys.*, 2010, **367**, 44-47.

4 A.K. van Helden, J.W. Jansen, A. Vrij, *J. Colloid Interf. Sci.*, 1981, **81**, 354-368.

5 E. Betzig, G.H. Patterson, R. Sougrat, O.W. Lindwasser, S. Olenych, J.S. Bonifacino, M.W. Davidson, J. Lippincott-Schwartz, H.F. Hess, *Science*, 2006, **313**, 1642-1645.

6 H. Deschout, F. Cella Zanacchi, M. Mlodzianoski, A. Diaspro, J. Bewersdorf, S.T. Hess K. Braeckmans, *Nat. Meth.*, 2014, **11**, 253-266.

7 S. Lou, X. Jiang, P.J. Scott, *Proc. R. Soc. A.*, 2013, **469**, 20130150.

8 C. Xu, S. Peng, G.G. Qiao, V. Gutowski, D. Lohse X. Zhang, *Soft Matter*, 2014, **10**, 7857-7864.
