# Supplementary figures and images for: iPAINT: a general approach tailored to image the topology of interfaces with nanometer resolution
Source: Nanoscale. 2016 Mar 30;8(16):8712–6. doi: 10.1039/c6nr00445h (PMC5050559; doi:10.1039/c6nr00445h)

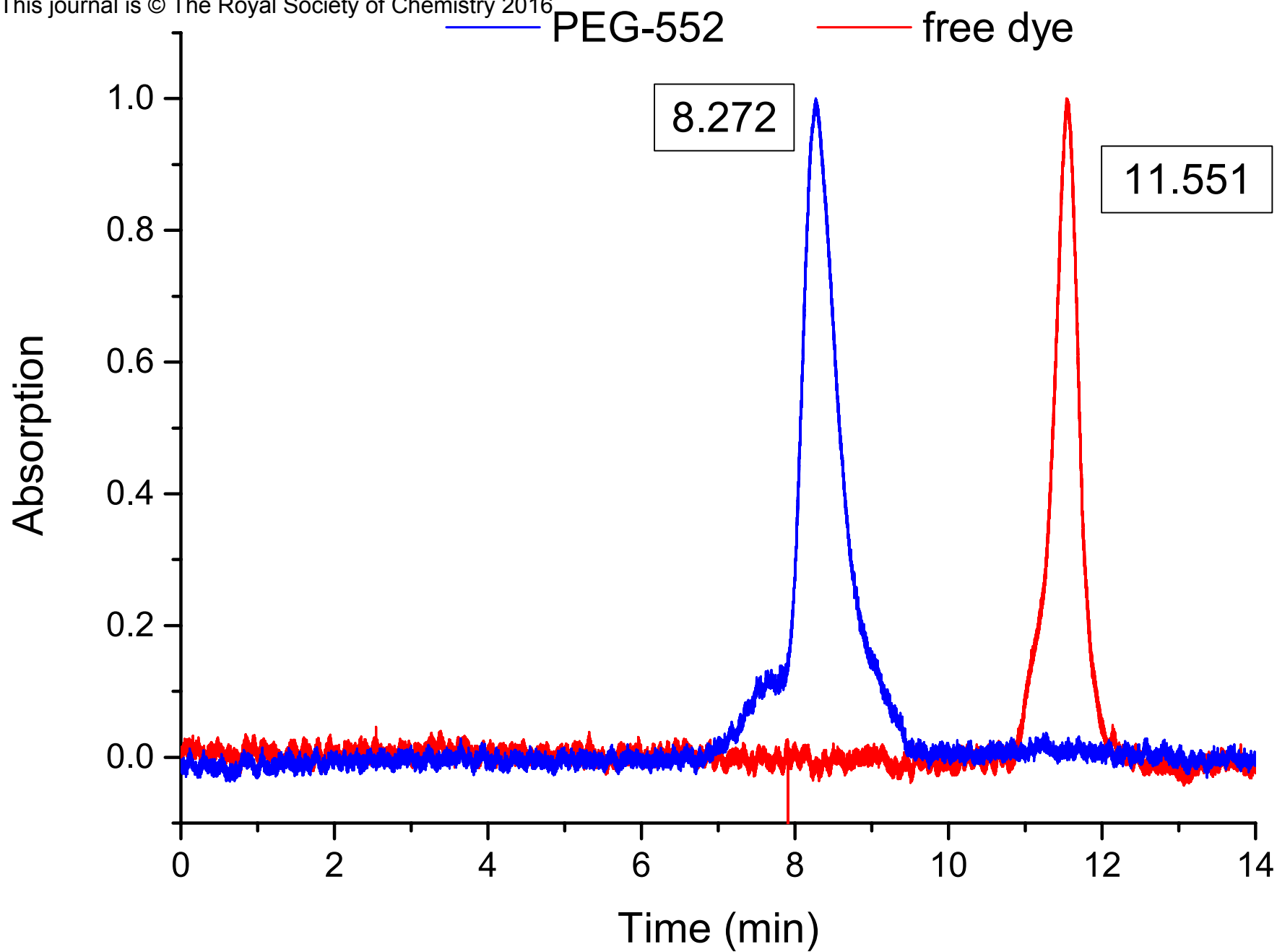

Supplement: Supplementary file 2 [file NR-008-C6NR00445H-s002.pdf]

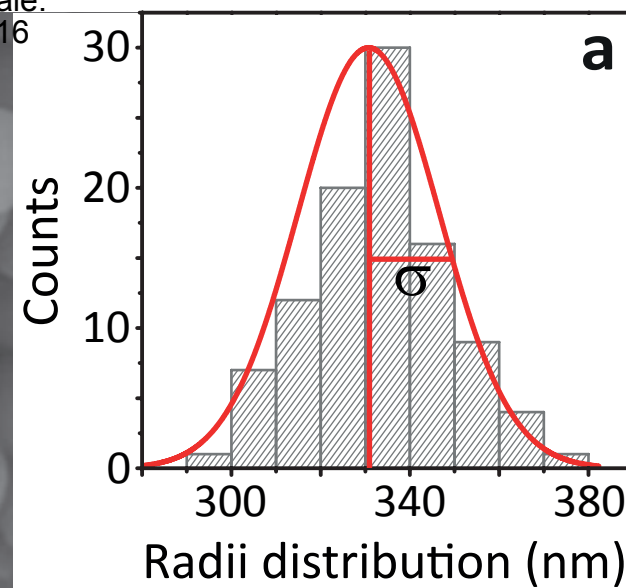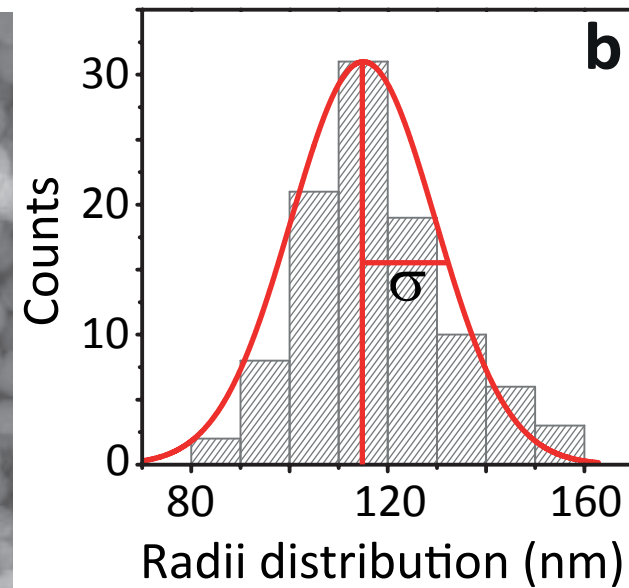

Supplement: Supplementary file 3 [file NR-008-C6NR00445H-s003.pdf]

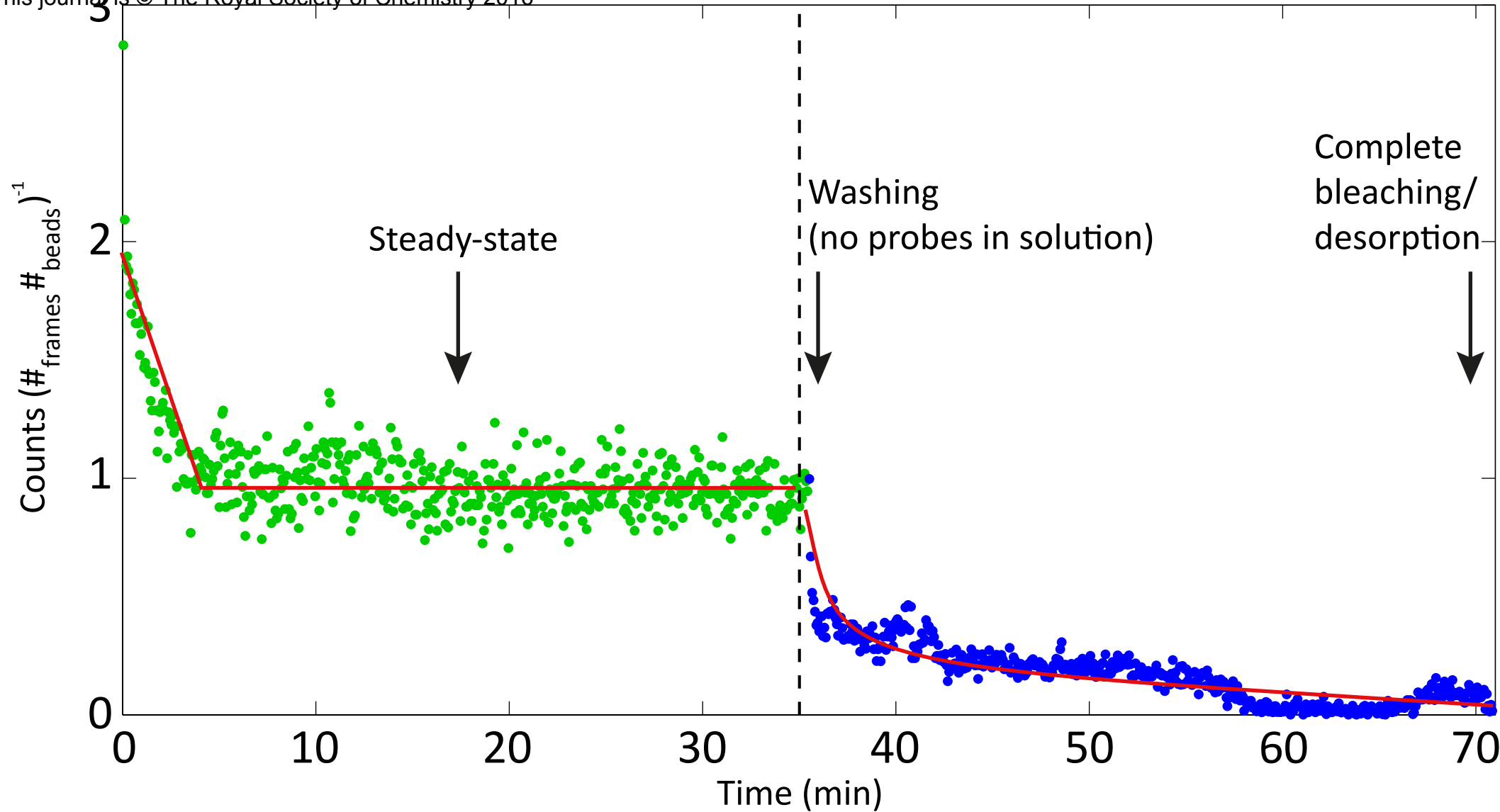

Supplement: Supplementary file 4 [file NR-008-C6NR00445H-s004.pdf]

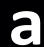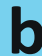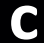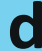

Supplement: Supplementary file 5 [file NR-008-C6NR00445H-s005.pdf]

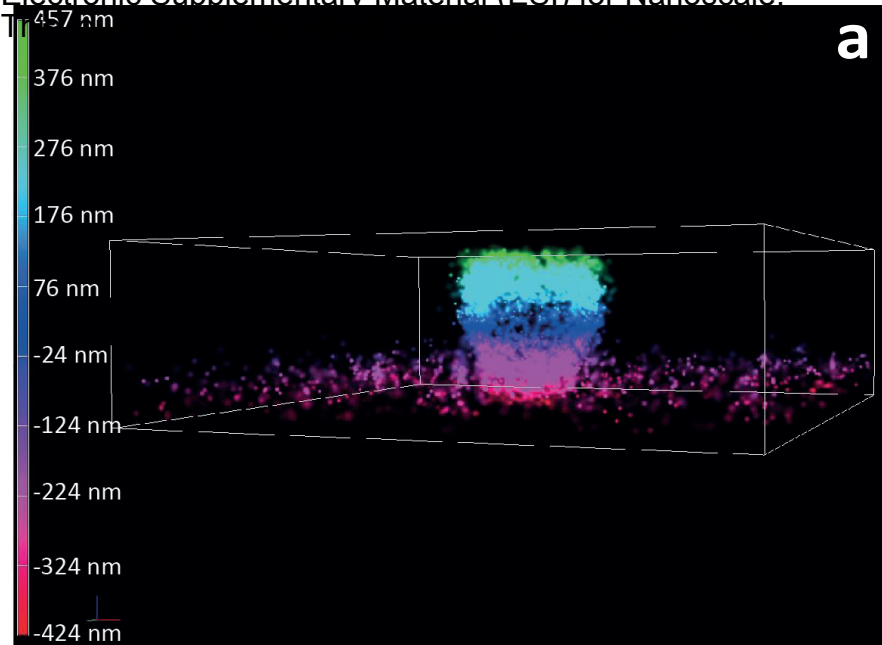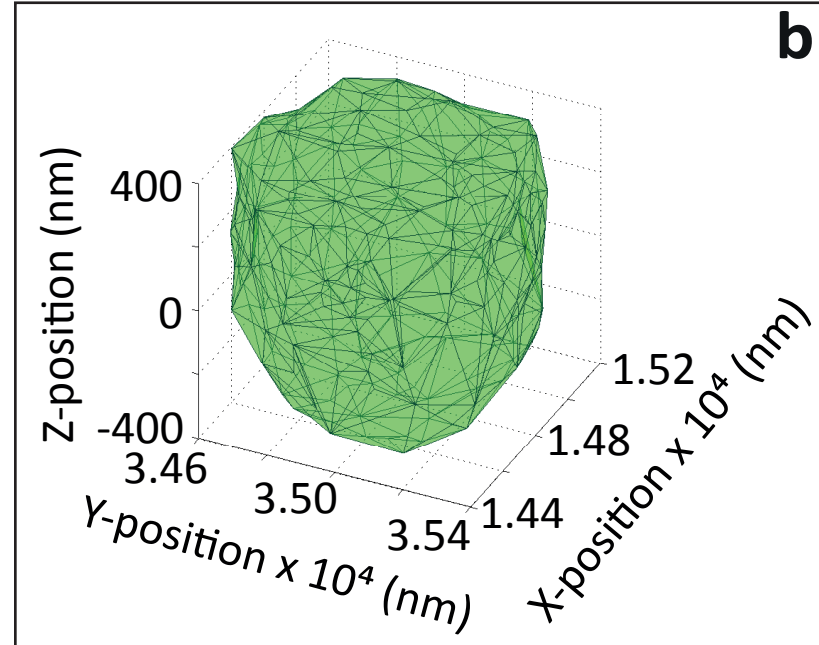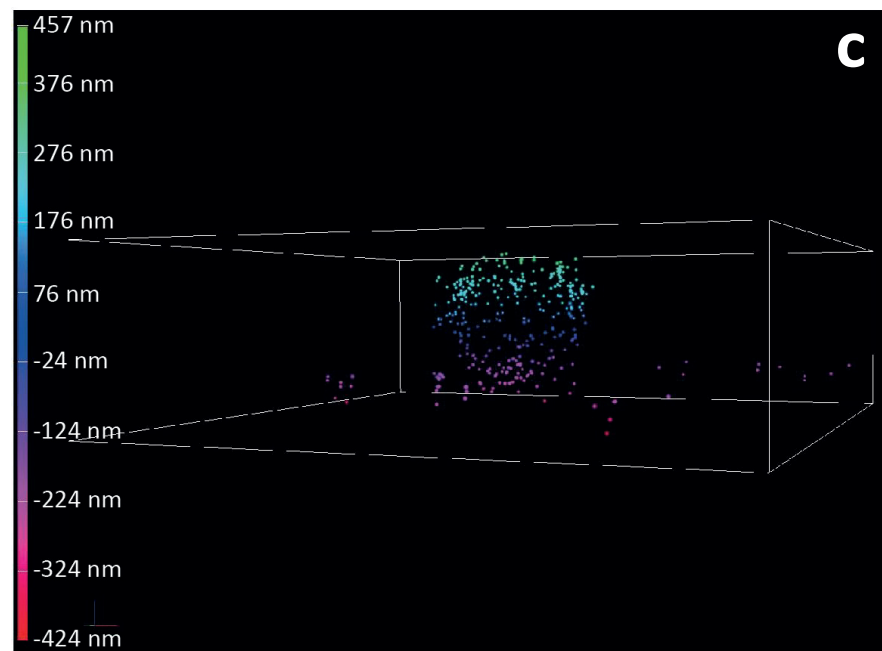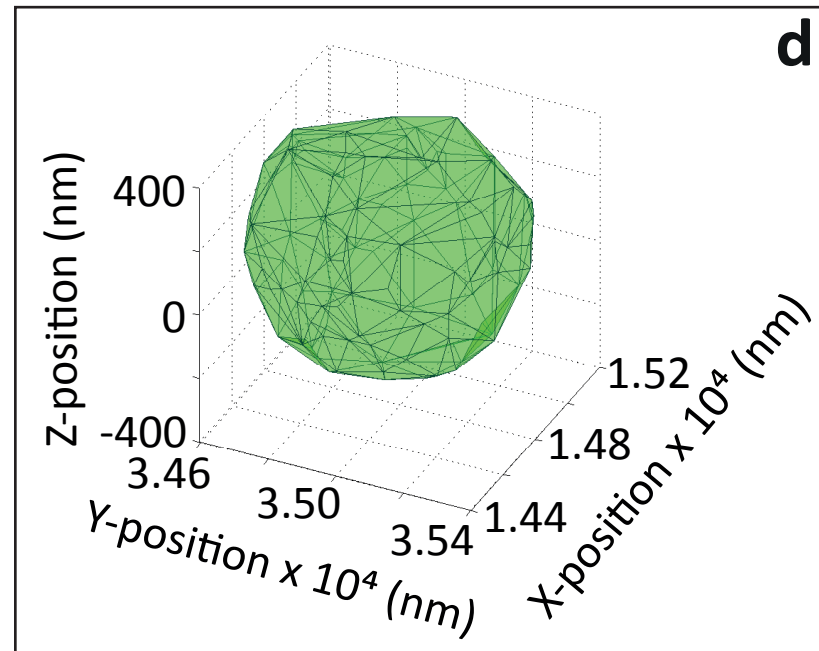

Supplement: Supplementary file 7 [file NR-008-C6NR00445H-s007.pdf]

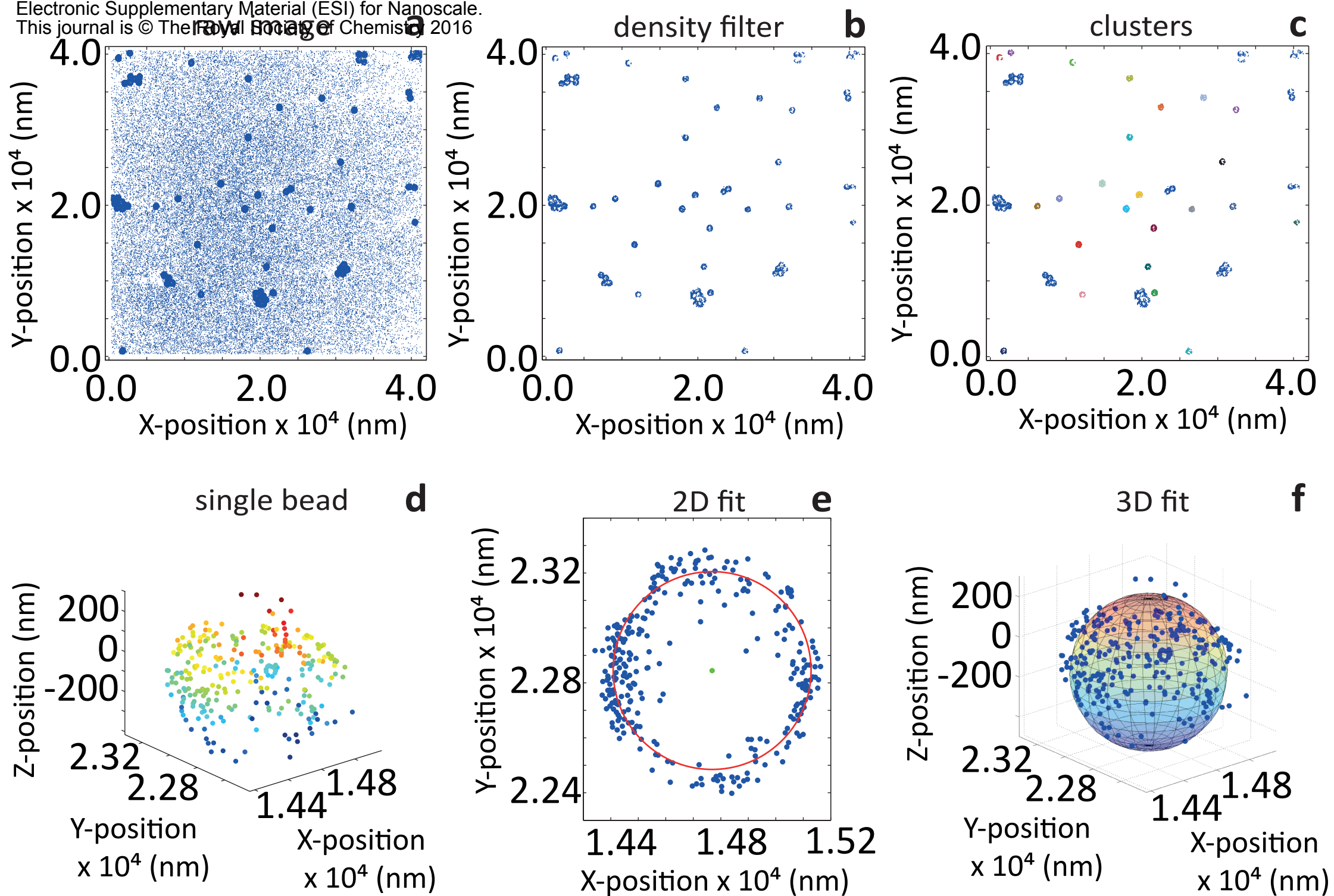

Supplement: Supplementary file 8 [file NR-008-C6NR00445H-s008.pdf]

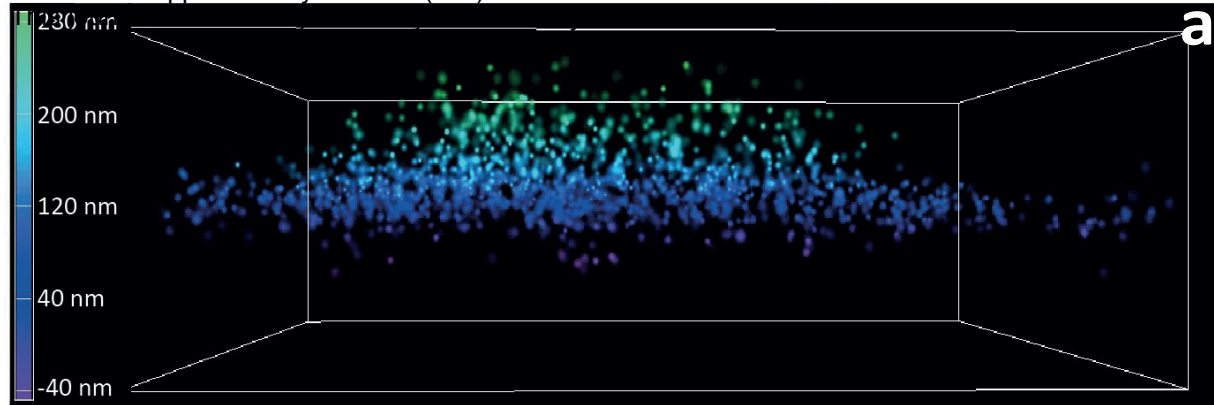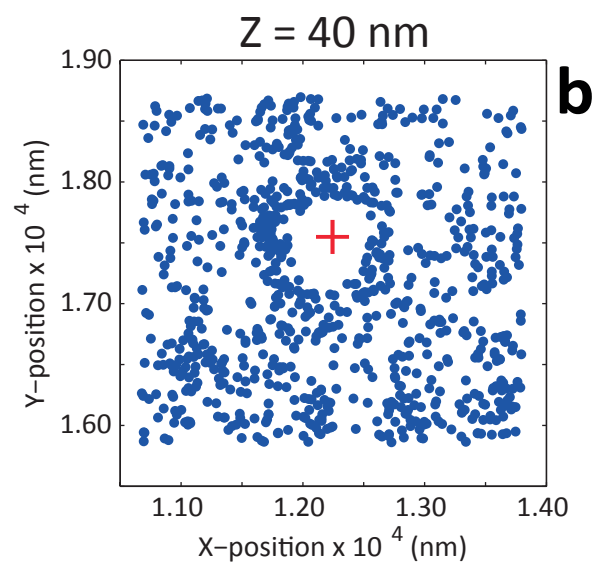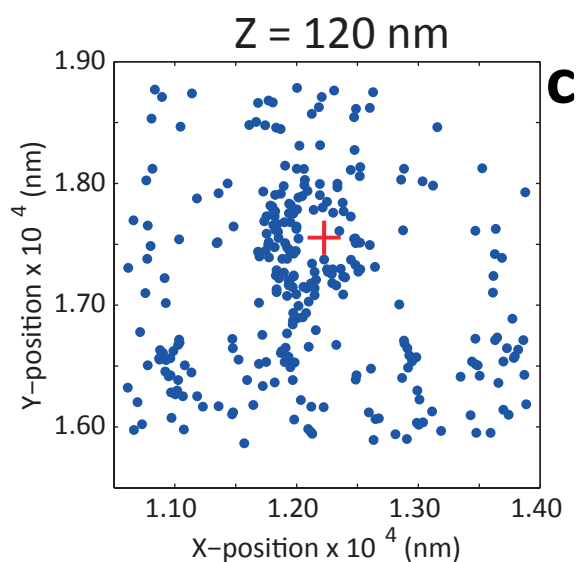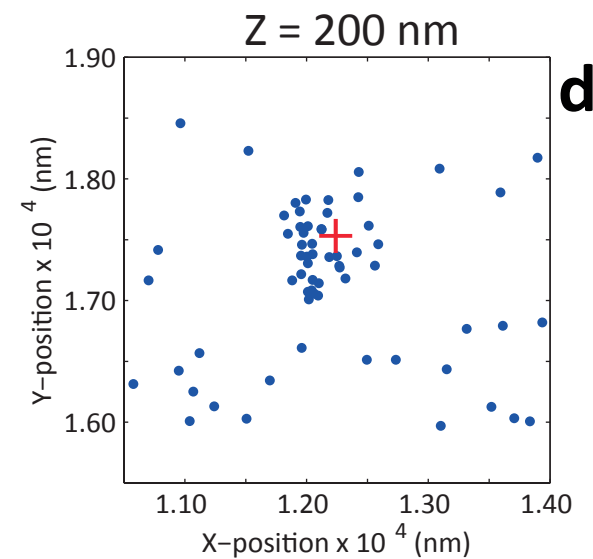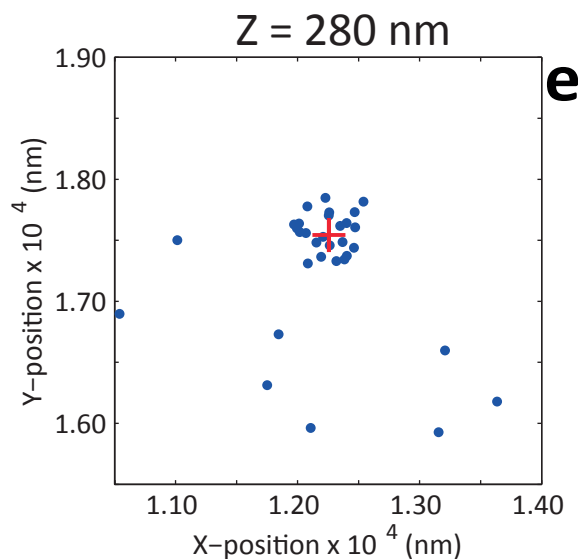

Supplement: Supplementary file 9 [file NR-008-C6NR00445H-s009.pdf]
