# Supplementary material for: iPAINT: a general approach tailored to image the topology of interfaces with nanometer resolution
Source: Nanoscale. 2016 Mar 30;8(16):8712–6. doi: 10.1039/c6nr00445h (PMC5050559; doi:10.1039/c6nr00445h)

Electronic Supplementary Material (ESI) for Nanoscale.
This journal is © The Royal Society of Chemistry 2016

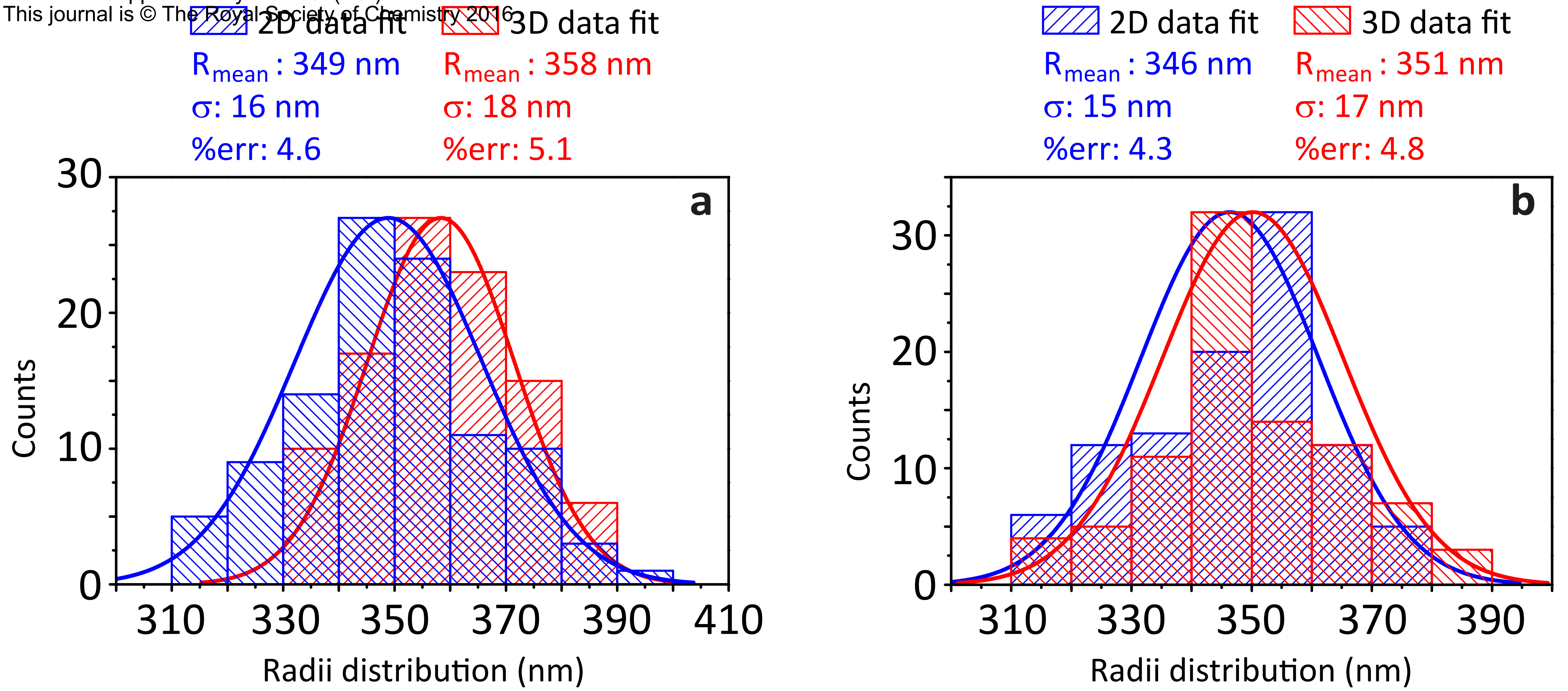

Supplement: Supplementary file 6 [file NR-008-C6NR00445H-s006.pdf]
